# Supplementary material for: Nucleic-acid-base photofunctional cocrystal for information security and antimicrobial applications
Source: Nat Commun. 2024 Mar 22;15:2561. doi: 10.1038/s41467-024-46869-6 (PMC10959985; doi:10.1038/s41467-024-46869-6)
Supplement: Supplementary file 1 — Supplementary Information [file 41467_2024_46869_MOESM1_ESM.pdf]

Supplementary Information

Nucleic-Acid-Base Photofunctional Cocrystal for Information Security and Antimicrobial Applications

Wenqing Xu‡[1,2,3], Guanheng Huang‡[1], Zhan Yang[4], Ziqi Deng[1], Chen Zhou[5], Jian-An Li[6], Ming-De Li[5]*, Tao Hu[2,3]*, Ben Zhong Tang[4]*, David Lee Phillips[1]*

[1] Department of Chemistry and State Key Laboratory of Synthetic Chemistry, The University of Hong Kong, Pokfulam Road, Hong Kong, 999077, China.

[2] State Key Laboratory of Oral Diseases & National Clinical Research Center for Oral Diseases, Sichuan University, Chengdu, Sichuan, 610041, China.

[3] Department of Preventive Dentistry, West China Hospital of Stomatology, Sichuan University, Chengdu, Sichuan, 610041, China.

[4] School of Science and Engineering, Shenzhen Institute of Aggregate Science and Technology, The Chinese University of Hong Kong, Shenzhen, Guangdong, 518172, China.

[5] Key Laboratory for Preparation and Application of Ordered Structural Materials of Guangdong Province, Department of Chemistry, Shantou University, Shantou, Guangdong, 515031, China.

[6] Sustainable Energy and Environment Thrust, The Hong Kong University of Science and Technology (Guangzhou), Nansha, Guangzhou, Guangdong, 510000, China.

‡These two authors contributed equally to this work.

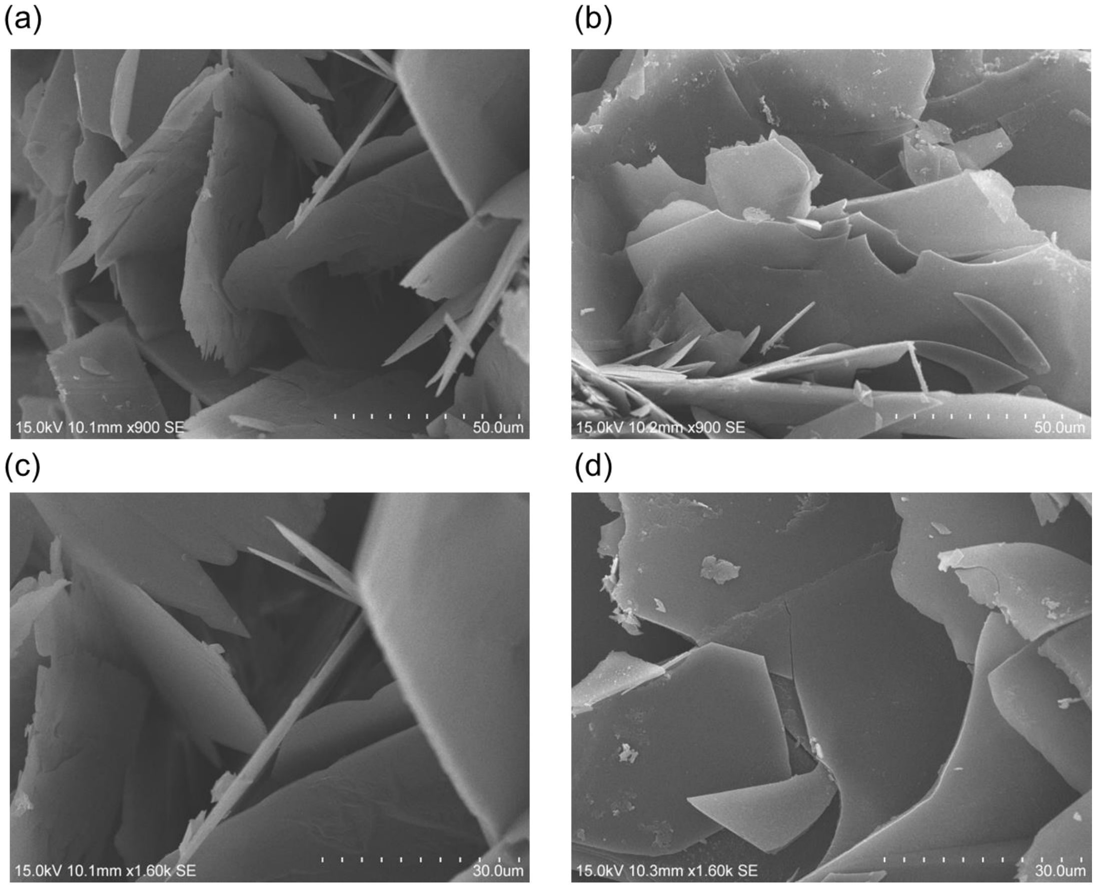

Supplementary Fig 1. SEM images of U-MA cocrystal at different magnifications. (a) and (b) are x900. (c) and (d) are x1.6k. U-MA cocrystal exhibits sheet-like structure.

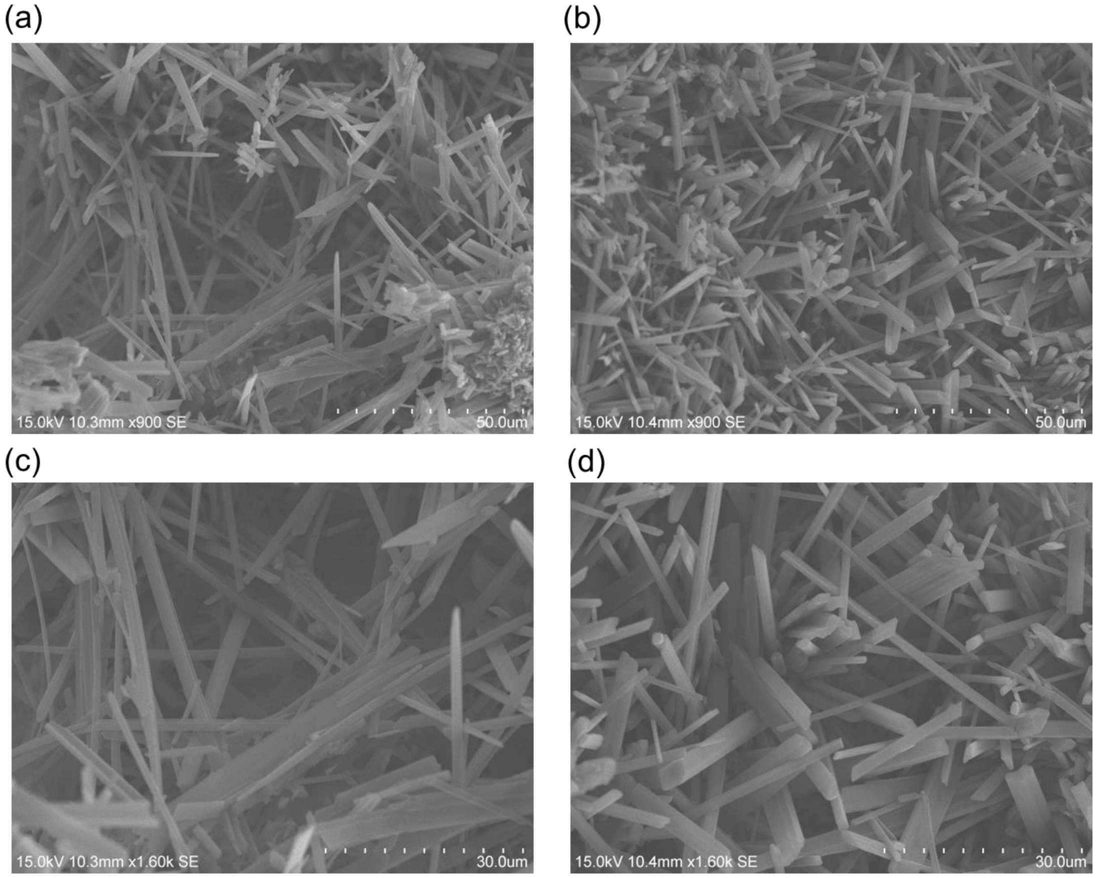

Supplementary Fig 2. SEM images of U-B cocrystal at different magnifications. (a) and (b) are x900. (c) and (d) are x1.6k. U-B cocrystal exhibits fine needle-like structure.

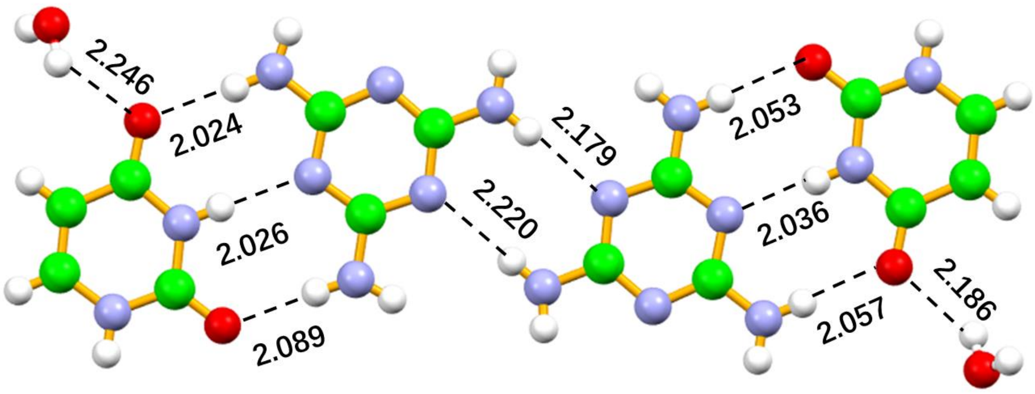

Supplementary Fig 3. The intermolecular hydrogen bond mode of U-MA single crystal structure.

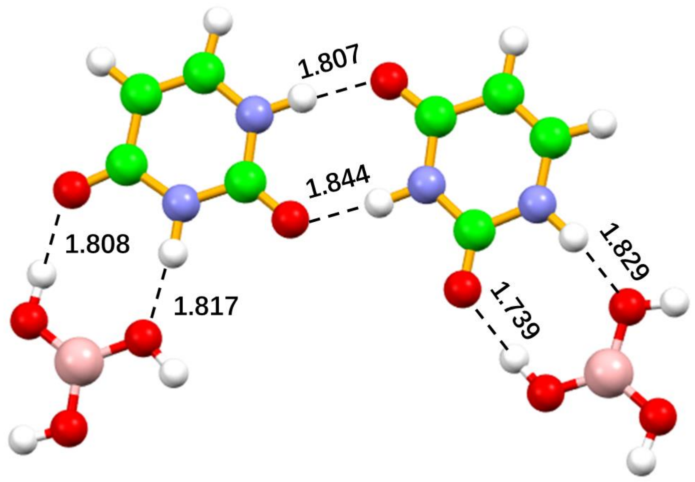

Supplementary Fig 4. The simulated intermolecular hydrogen bond mode of U-B.

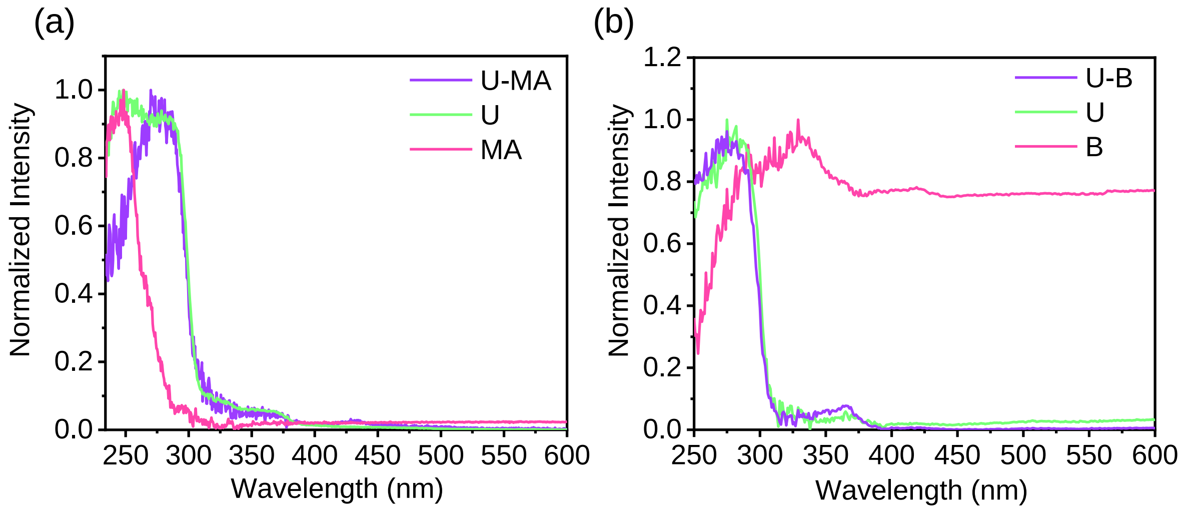

Supplementary Fig 5. (a) The UV-Vis spectrum of U, MA, and U-MA powders. **(b)** The UV-Vis spectrum of U, Boric acid, and U-B powders.

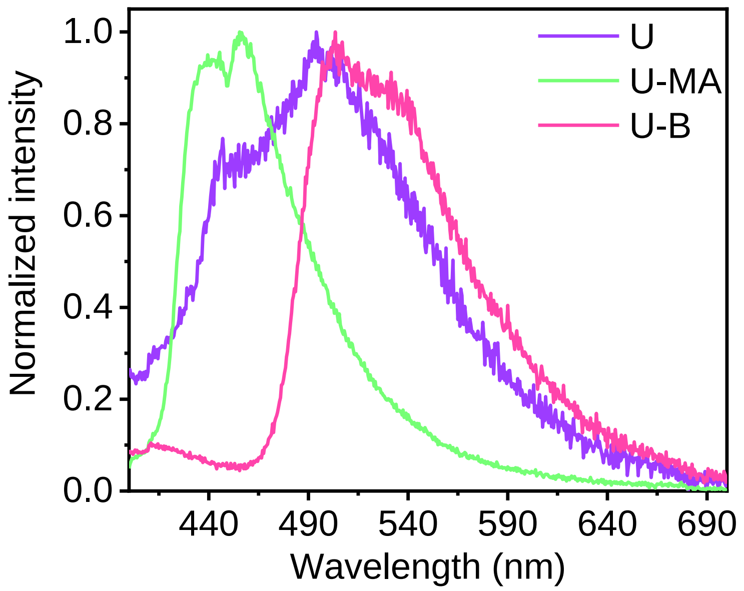

Supplementary Fig 6. The normalized delay photoluminescence spectrum (5 ms delay) of U, U-MA, and U-B powders upon 365 nm excitation.

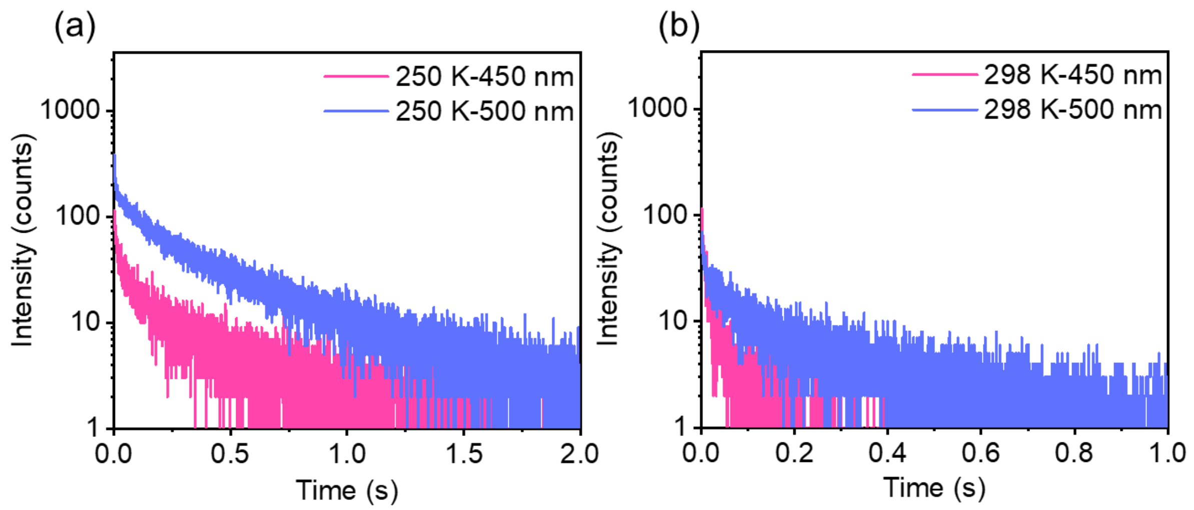

Supplementary Fig 7. The comparison of phosphorescent kinetic decay of U at 450 nm and 500 nm at **(a)** 250 K. **(b)** 298 K.

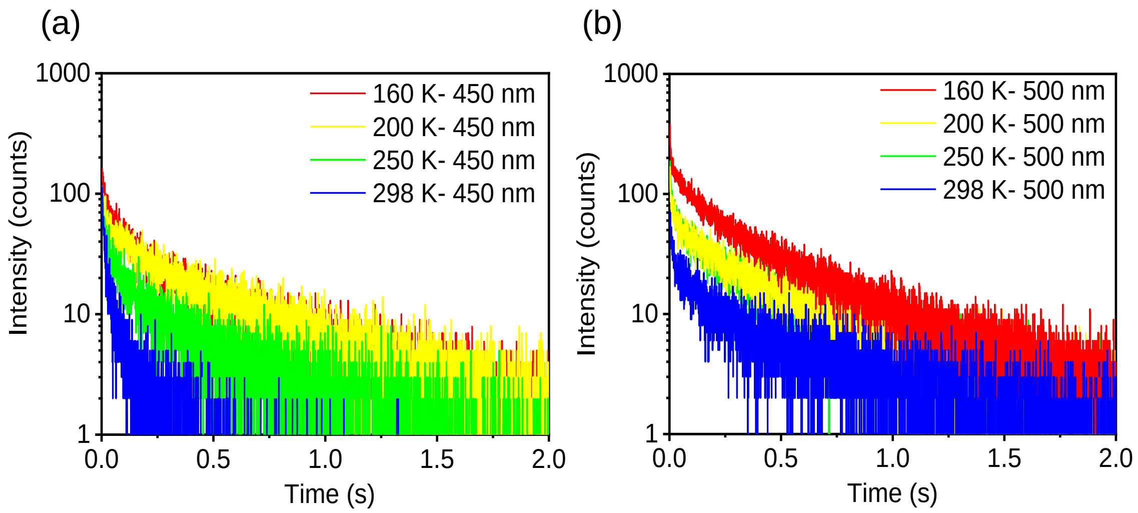

Supplementary Fig 8. The phosphorescent kinetic of U under vacuum conditions at different temperatures **(a)** at 450 nm. **(b)** at 500 nm.

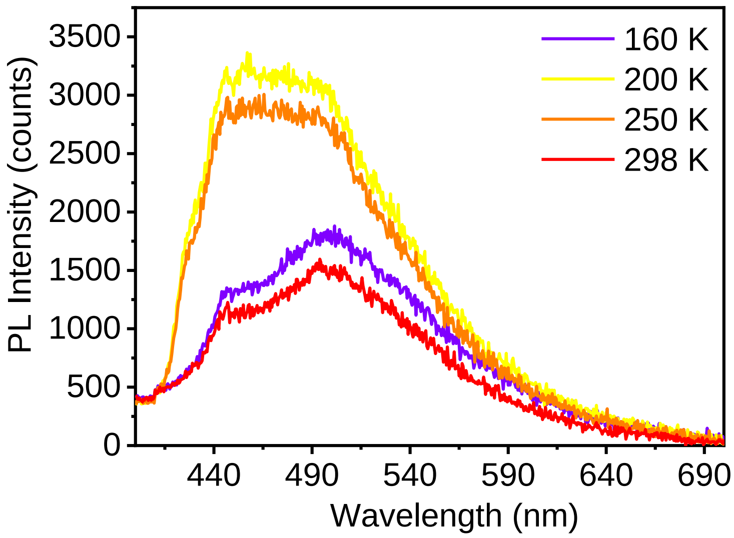

Supplementary Fig 9. Phosphorescence spectra of the U under vacuum conditions at different temperatures upon 365 nm excitation.

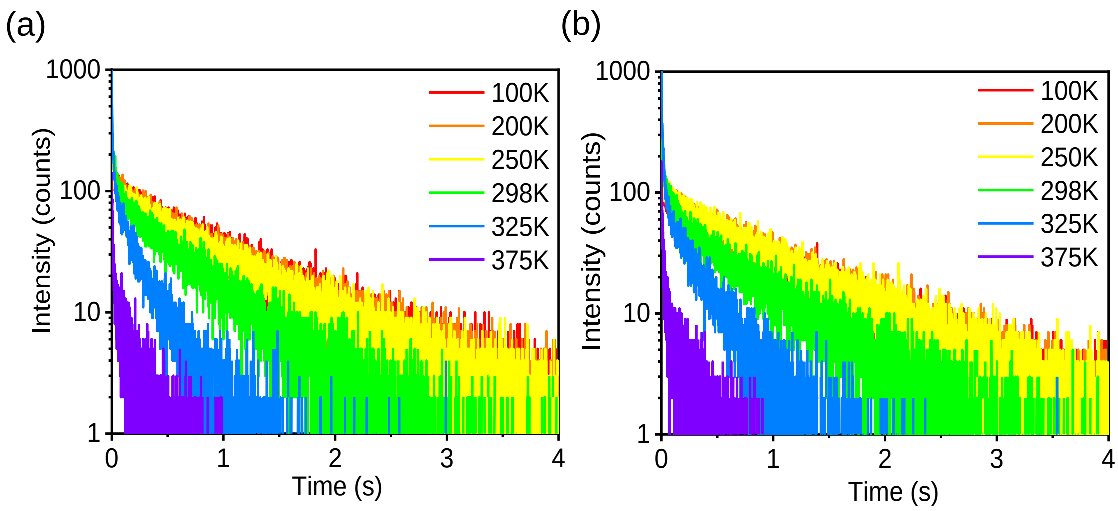

Supplementary Fig 10. The phosphorescent kinetic of U-MA under vacuum conditions at different temperatures **(a)** at 443 nm. **(b)** at 460 nm.

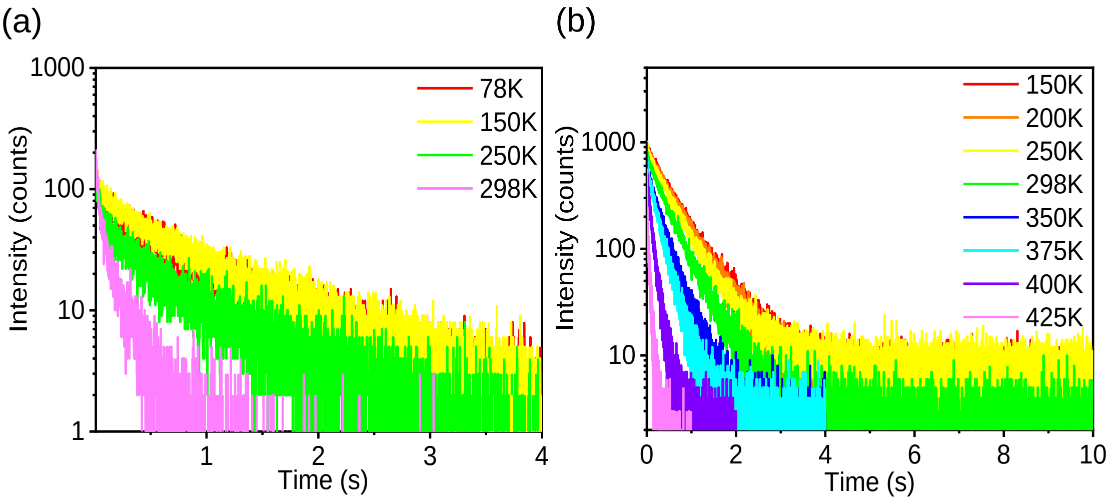

Supplementary Fig 11. The phosphorescent kinetic of U-B under vacuum conditions at different temperatures **(a)** at 453 nm. **(b)** at 536 nm.

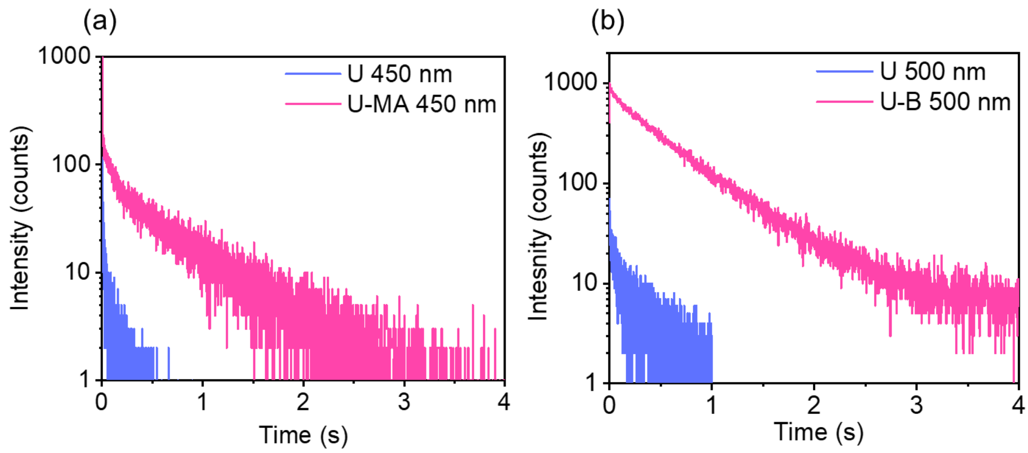

Supplementary Fig 12. The comparison of phosphorescent kinetic decay of (a) U and U-MA at 450 nm (b) U and U-B at 500 nm.

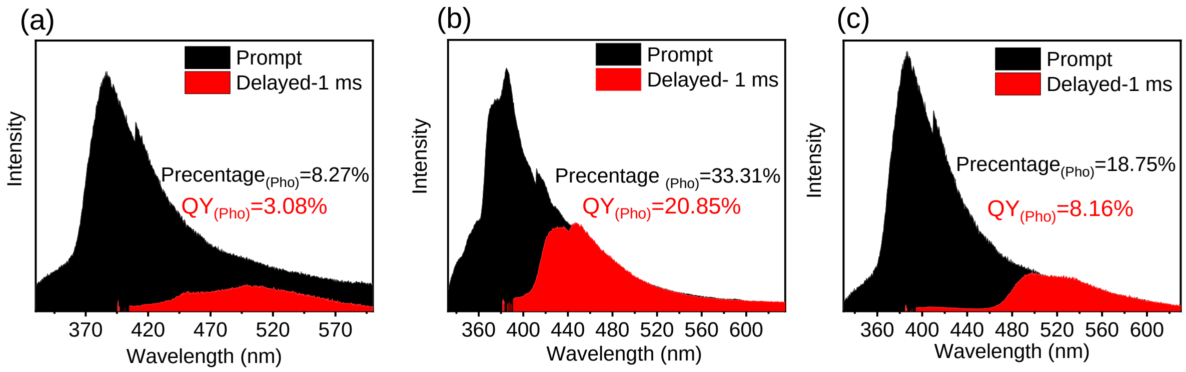

Supplementary Fig 13. The absolute quantum yield and phosphorescence quantum yield of **(a)** U. **(b)** U-MA. **(c)** U-B.

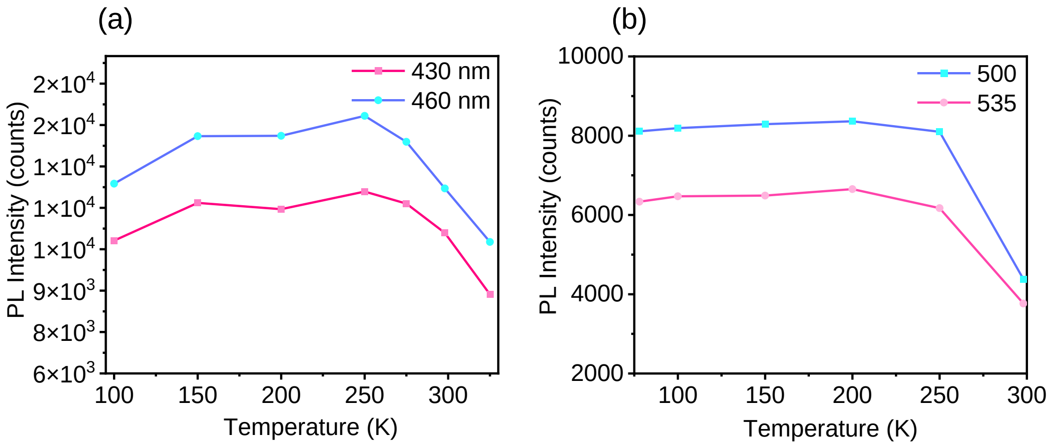

Supplementary Fig 14. The selected phosphorescence intensity as a function of the temperature of **(a)** U-MA. **(b)** U-B.

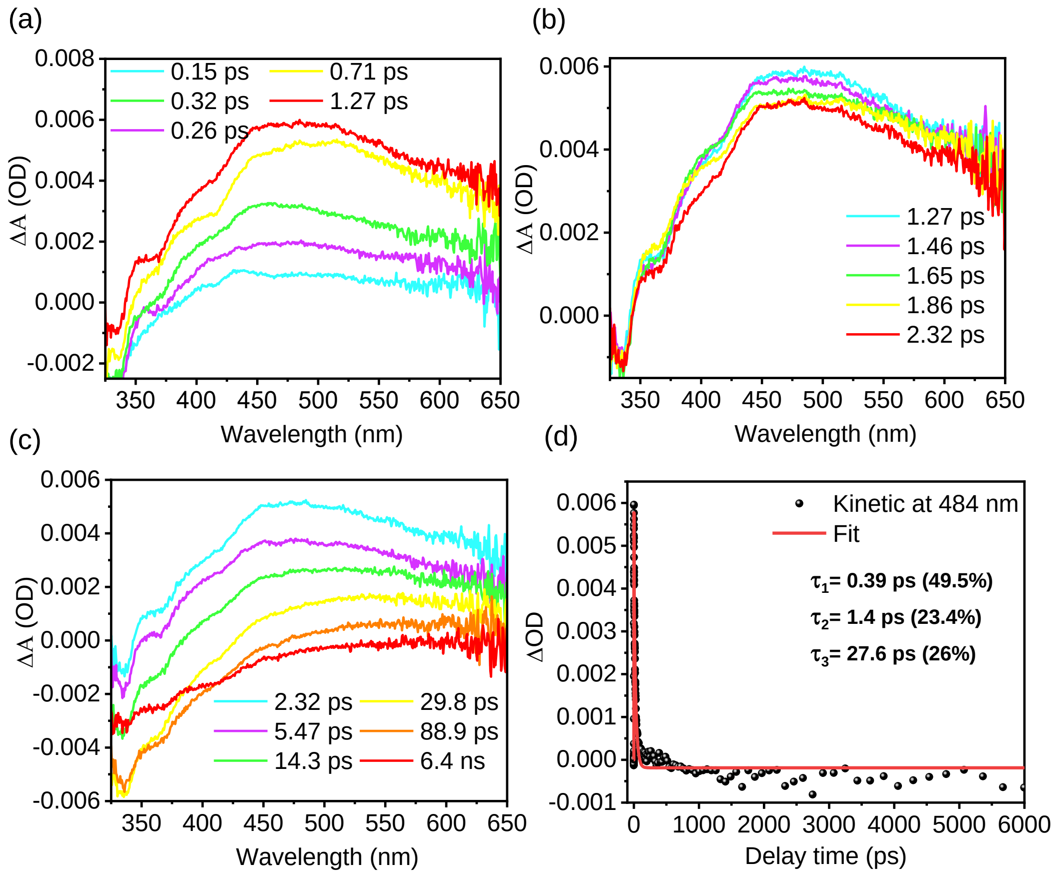

Supplementary Fig 15. fs-TA spectra of U film under 290 nm excitation **(a)** from 0.15 to 1.27 ps. **(b)** from 1.27 to 2.32 ps. **(c)** from 2.32 ps to 6.4 ns **(d)** Kinetic fitting for U at 484 nm.

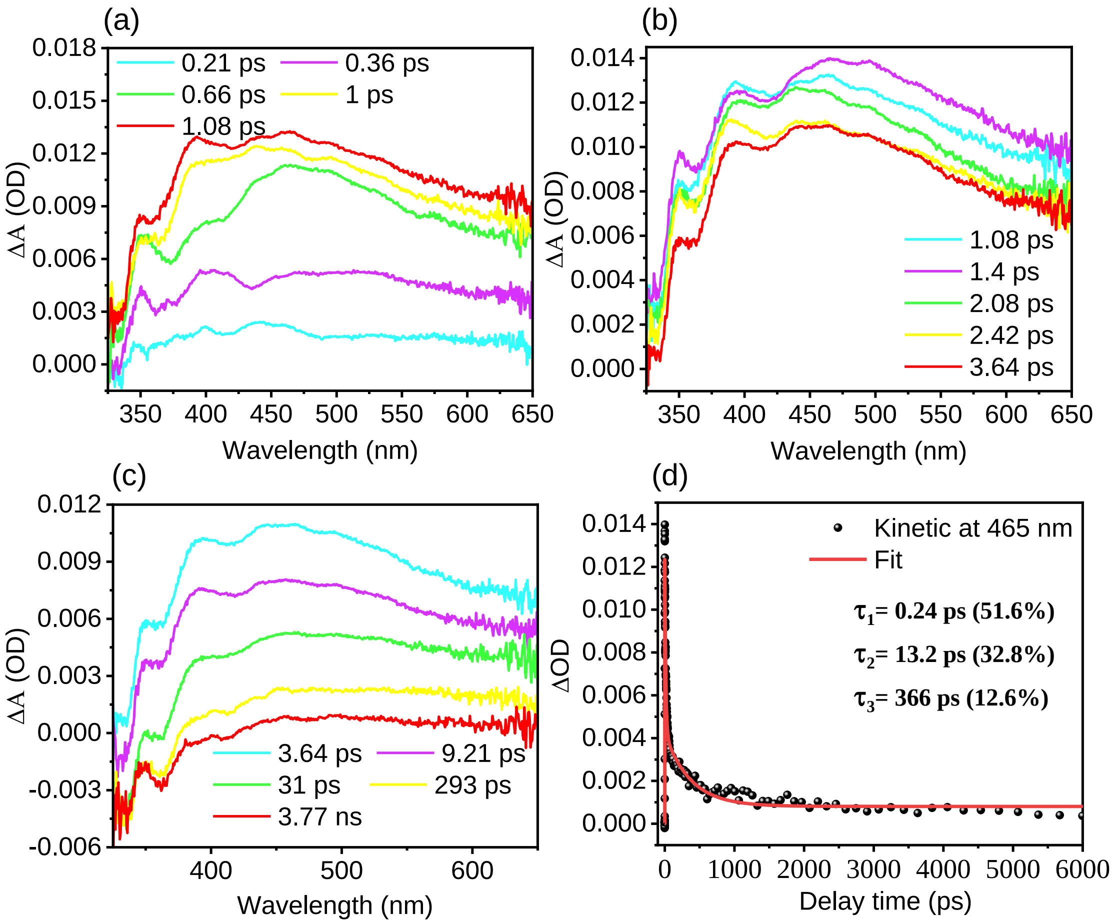

Supplementary Fig 16. fs-TA spectra of U-B film under 290 nm excitation **(a)** from 0.21 to 1.08 ps. **(b)** from 1.08 to 3.64 ps. **(c)** from 3.64 ps to 3.77 ns. **(d)** Kinetic fitting for U-B at 465 nm.

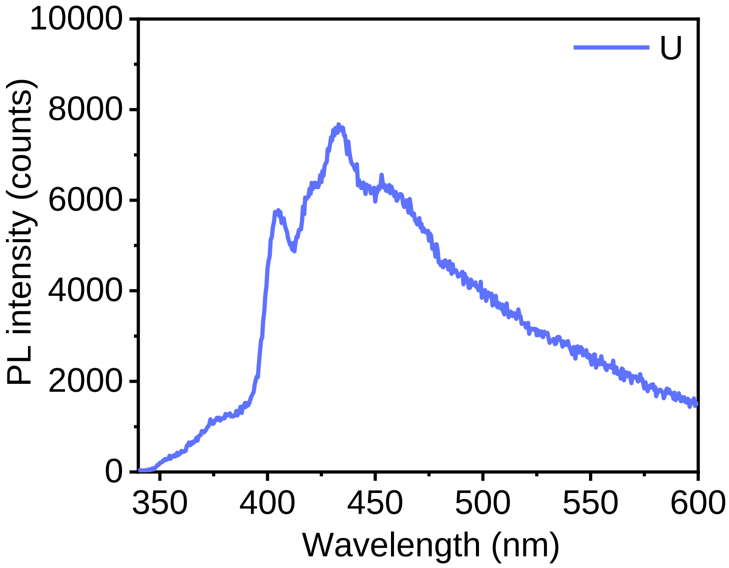

Supplementary Fig 17. The phosphorescence spectrum of U at 77 K in diluted methanol solution.

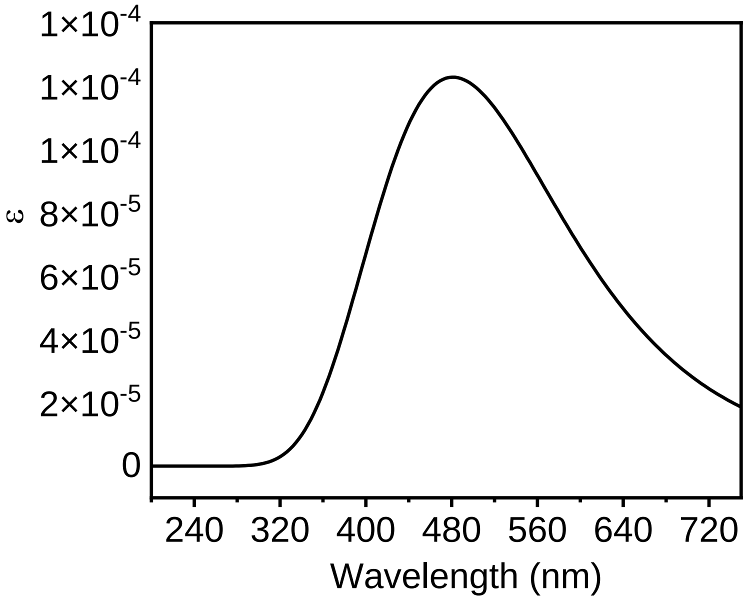

Supplementary Fig 18. The simulated phosphorescence spectrum of U monomer.

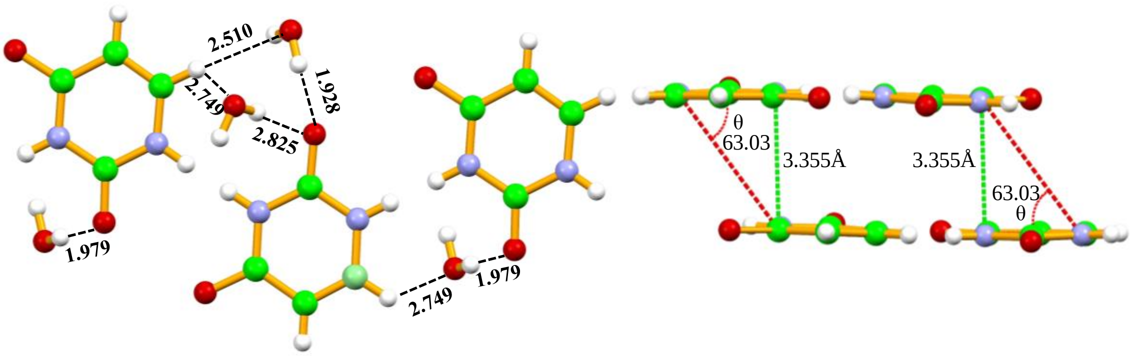

Supplementary Fig 19. The single crystal structure of U.

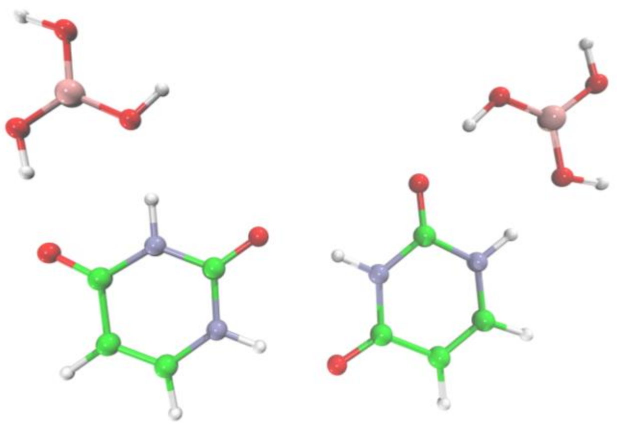

Supplementary Fig 20. The optimized geometry of the U-B.

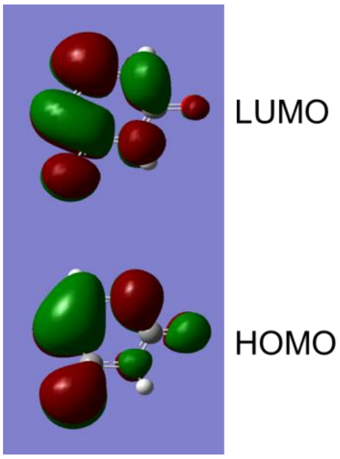

Supplementary Fig 21. The molecular orbital of U.

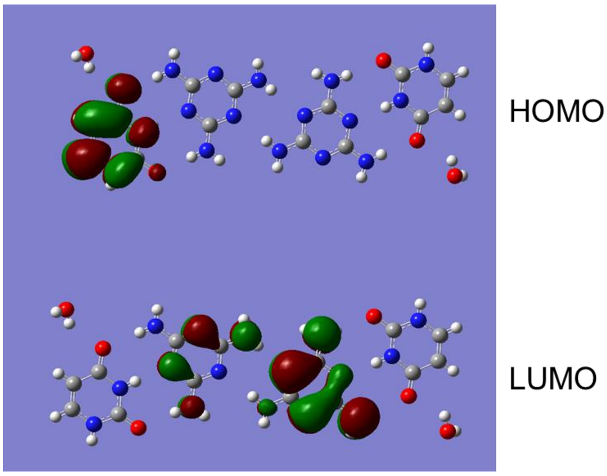

Supplementary Fig 22. The molecular orbital of U-MA.

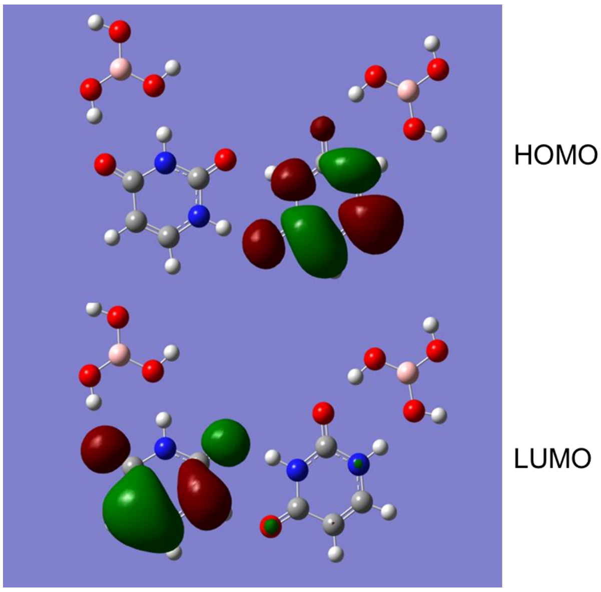

Supplementary Fig 23. The molecular orbital of U-B.

Microorganism	U-MA (μM)	U-B (μM)
Streptococcus mutans (*S. mutans*)	0.63	1.25
Staphylococcus aureus (S. aureus)	0.63	0.63
Escherichia coli (*E. coli*)	1.25	1.25
Candida albicans (*C. albicans*)	5.00	5.00

Supplementary Table 1. The minimum inhibitory concentration (MIC) of U-MA and U-B against microorganisms after exposure to light.

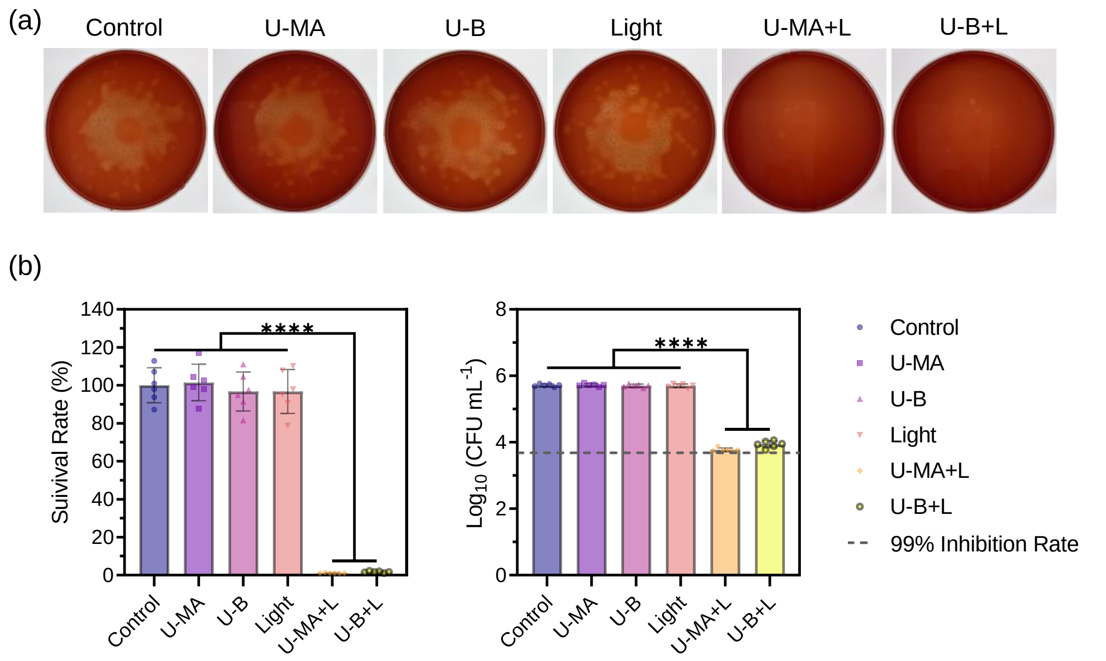

Supplementary Fig 24. Photodynamic killing of planktonic *S. mutans* by U-MA and U-B. **(a)** Representative images of blood agar plates for quantification of *S. mutans* viability. **(b)** Antibacterial efficiency of PDT mediated by U-MA and U-B against *S. mutans*. Error bars = Standard Deviation (n=6 biologically independent samples). One-way ANOVA was performed followed by Tukey's multiple comparisons. ****: $p < 0.0001$. L: Light.

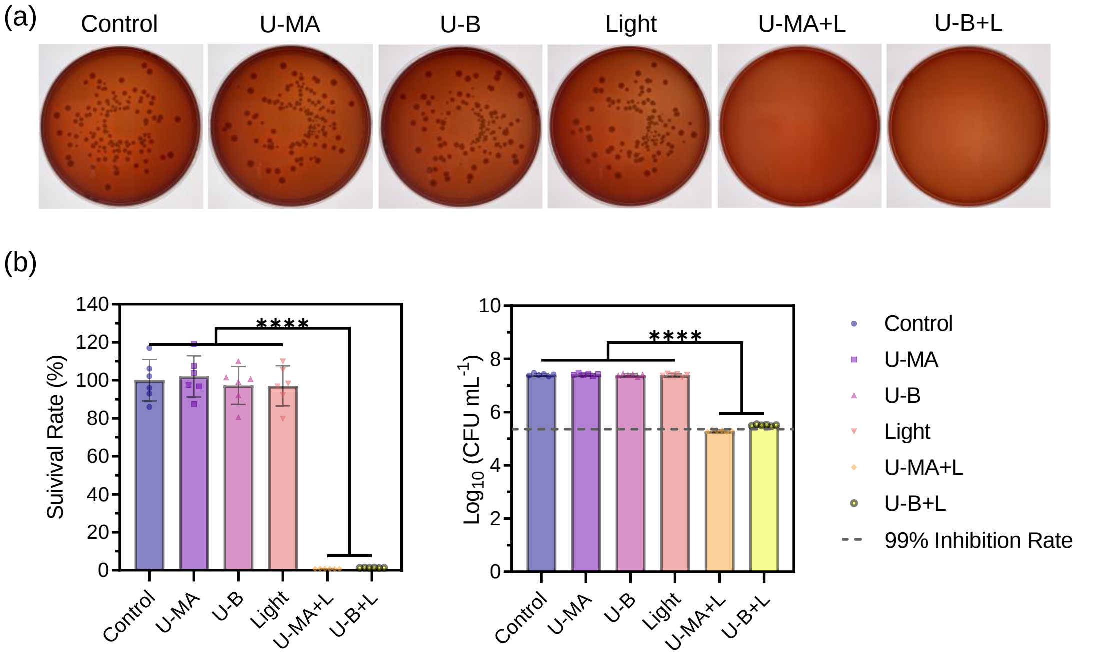

Supplementary Fig 25. Photodynamic killing of planktonic *S. aureus* by U-MA and U-B. **(a)** Representative images of blood agar plates for quantification of *S. aureus* viability. **(b)** Antibacterial efficiency of PDT mediated by U-MA and U-B against *S. aureus*. Error bars = Standard Deviation (n=6 biologically independent samples). One-way ANOVA was performed followed by Tukey's multiple comparisons. ****: $p < 0.0001$. L: Light.

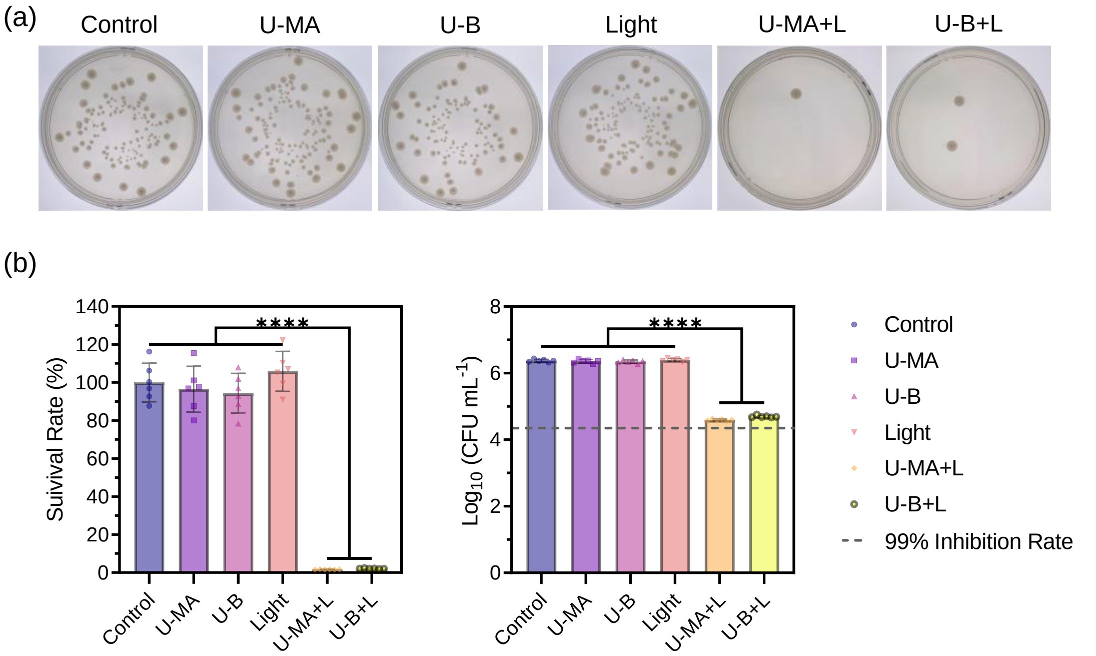

Supplementary Fig 26. Photodynamic killing of planktonic *E. coli* by U-MA and U-B. **(a)** Representative images of LB agar plates for quantification of *E. coli* viability. **(b)** Antibacterial efficiency of PDT mediated by U-MA and U-B against *E. coli*. Error bars = Standard Deviation (n=6 biologically independent samples). One-way ANOVA was performed followed by Tukey's multiple comparisons. ****: $p < 0.0001$. L: Light.

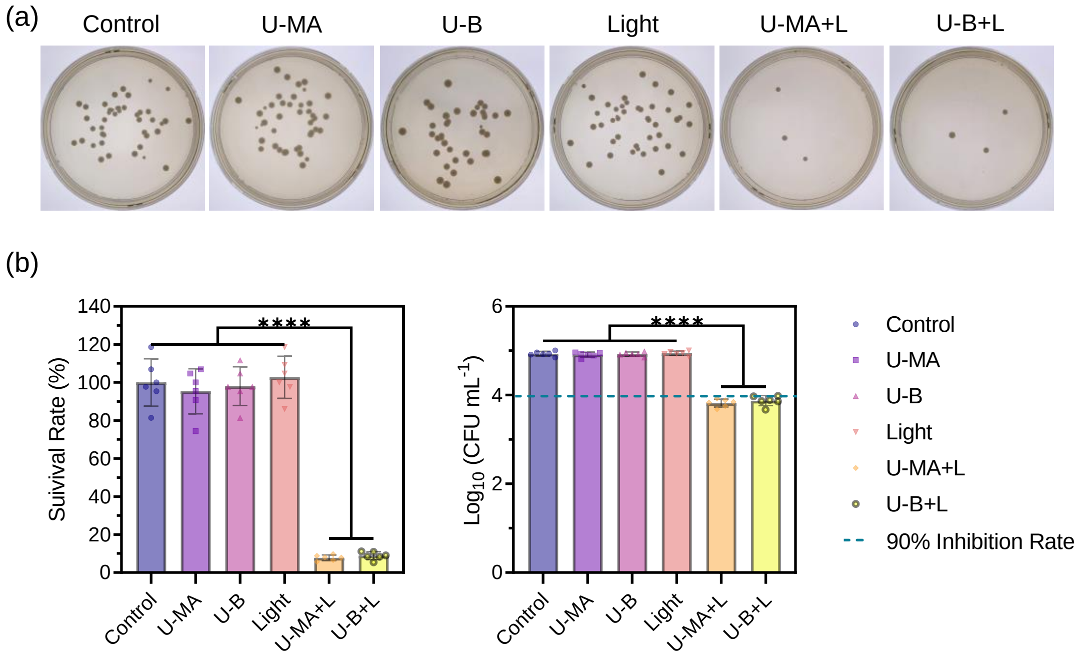

Supplementary Fig 27. Photodynamic killing of planktonic *C. albicans* by U-MA and U-B. **(a)** Representative images of SDA plates for quantification of *C. albicans* viability. **(b)** Antimicrobial efficiency of PDT mediated by U-MA and U-B against *C. albicans*. Error bars = Standard Deviation (n=6 biologically independent samples). One-way ANOVA was performed followed by Tukey's multiple comparisons. ****: $p < 0.0001$. L: Light.

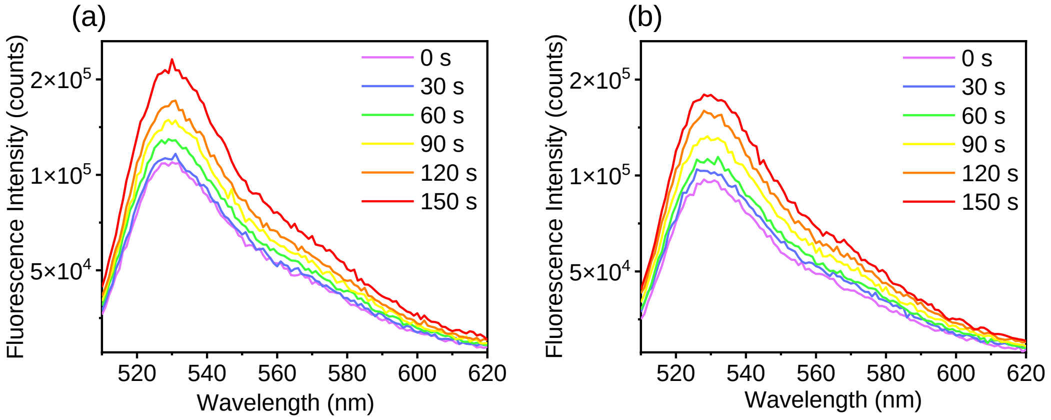

Supplementary Fig 28. Fluorescence response of SOSG upon treatment with **(a)** U-MA and **(b)** U-B under excitation at 365 nm for singlet oxygen generation, $\lambda ex = 504$ nm.

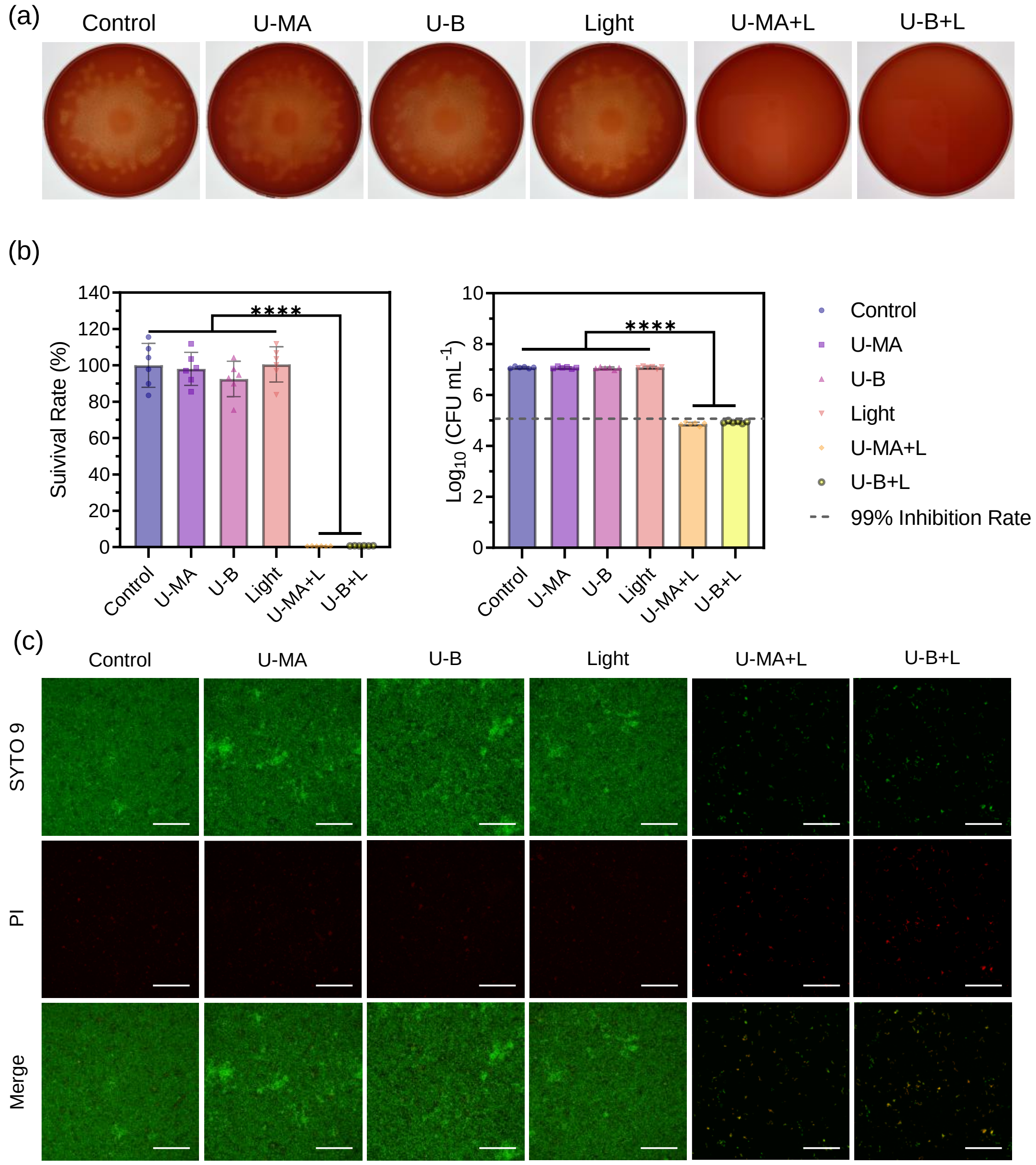

Supplementary Fig 29. Photodynamic inhibition of the formation of *S. mutans* biofilm by U-MA and U-B. *S. mutans* were cultured for 24 hours after treatment. **(a)** Representative images of blood agar plates for quantification of *S. mutans* viability. **(b)** Antibacterial efficiency of PDT mediated by U-MA and U-B against *S. mutans*. Error bars = Standard Deviation (n=6 biologically independent samples). One-way ANOVA was performed followed by Tukey's multiple comparisons. ****: $p < 0.0001$. **(c)** *S. mutans* biofilm detected by CLSM. The scale bars are 50 μm. L: Light.

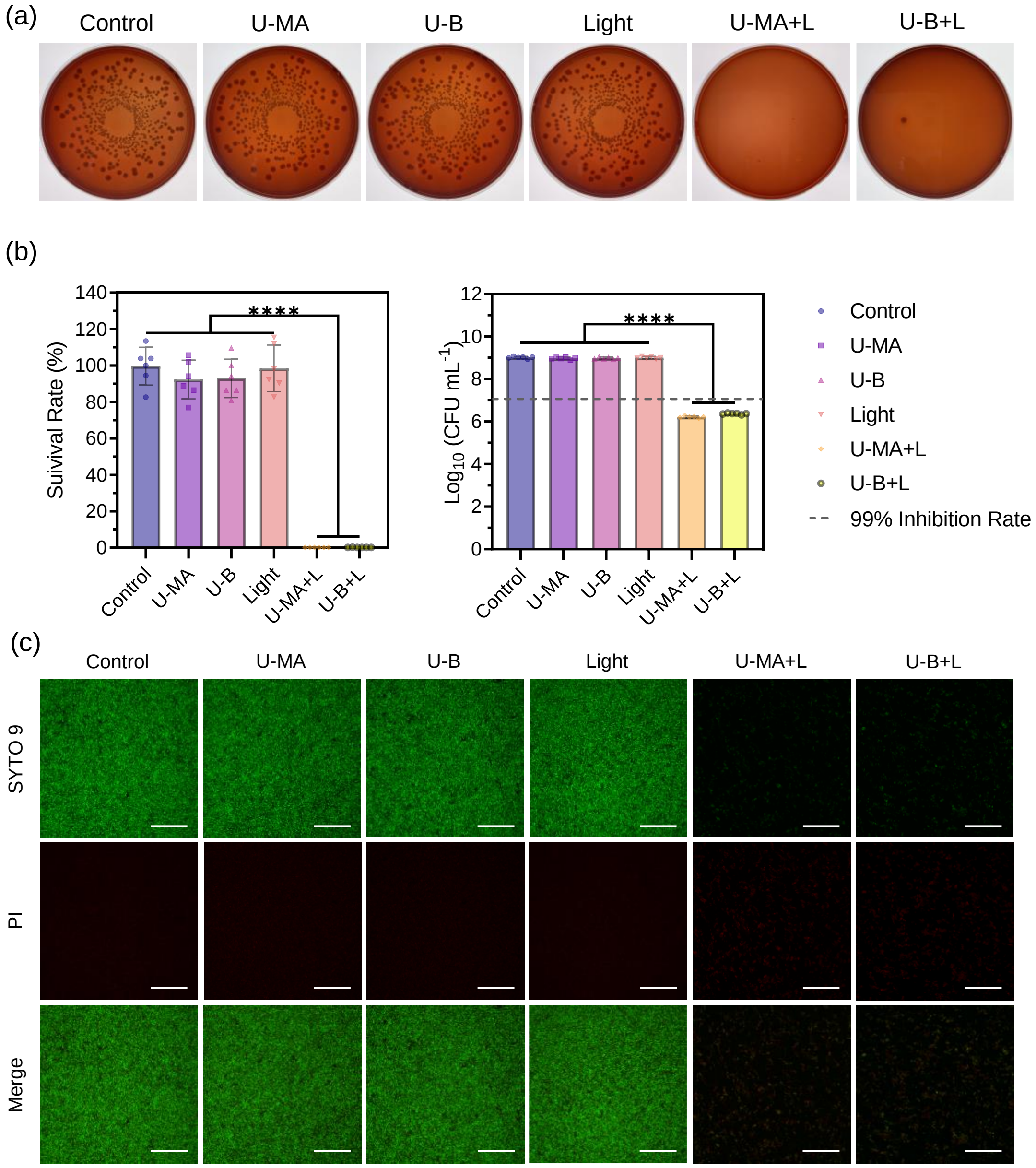

Supplementary Fig 30. Photodynamic inhibition of the formation of *S. aureus* biofilm by U-MA and U-B. *S. aureus* were cultured for 24 hours after treatment. **(a)** Representative images of blood agar plates for quantification of *S. aureus* viability. **(b)** Antibacterial efficiency of PDT mediated by U-MA and U-B against *S. aureus*. Error bars = Standard Deviation (n=6 biologically independent samples). One-way ANOVA was performed followed by Tukey's multiple comparisons. ****: $p < 0.0001$. **(c)** *S. aureus* biofilm detected by CLSM. The scale bars are 50 μm. L: Light.

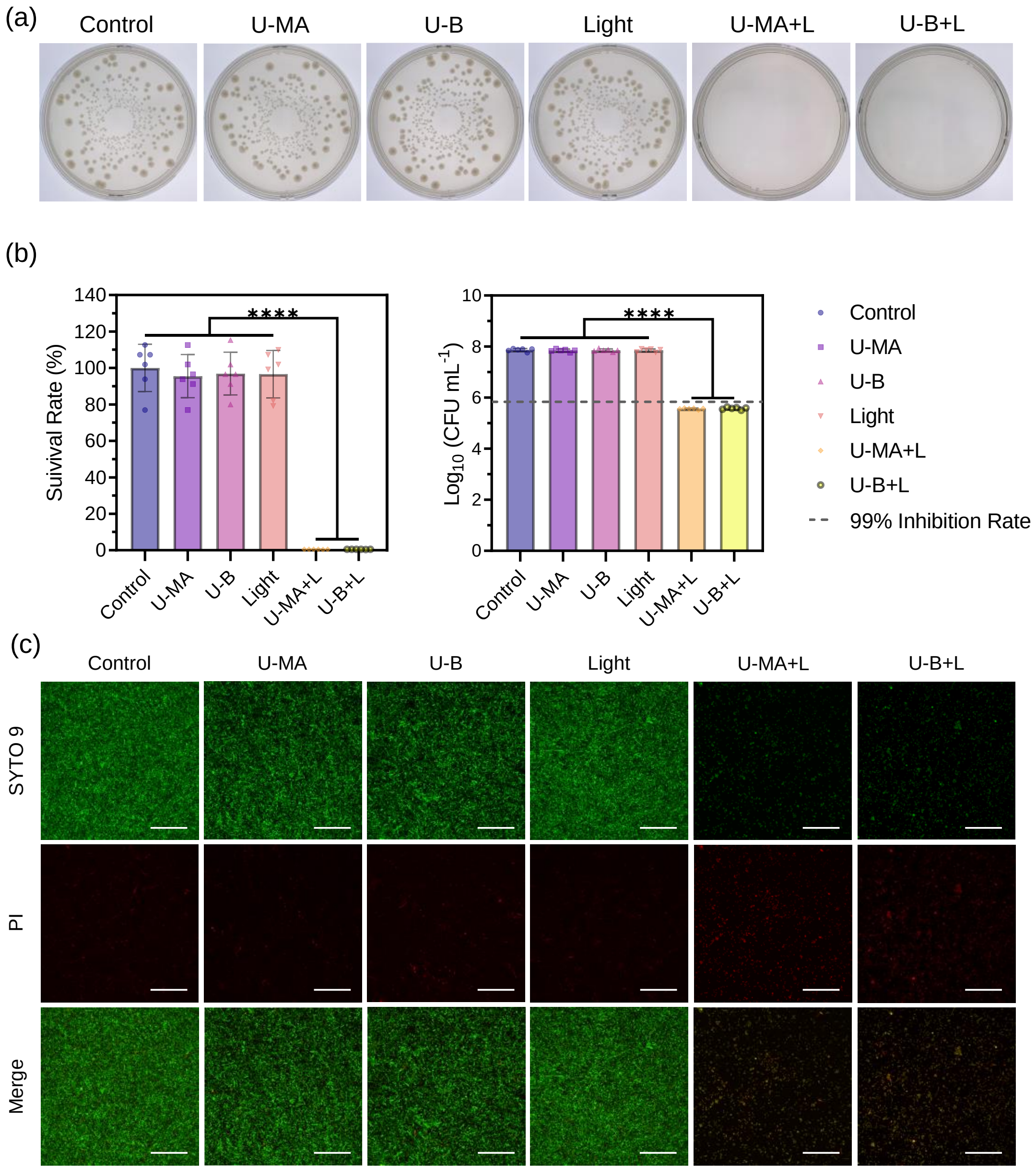

Supplementary Fig 31. Photodynamic inhibition of the formation of *E. coli* biofilm by U-MA and U-B. *E. coli* were cultured for 24 hours after treatment. **(a)** Representative images of LB agar plates for quantification of *E. coli* viability. **(b)** Antibacterial efficiency of PDT mediated by U-MA and U-B against *E. coli*. Error bars = Standard Deviation (n=6 biologically independent samples). One-way ANOVA was performed followed by Tukey's multiple comparisons. ****: $p < 0.0001$. **(c)** *E. coli* biofilm detected by CLSM. The scale bars are 50 μm. L: Light.

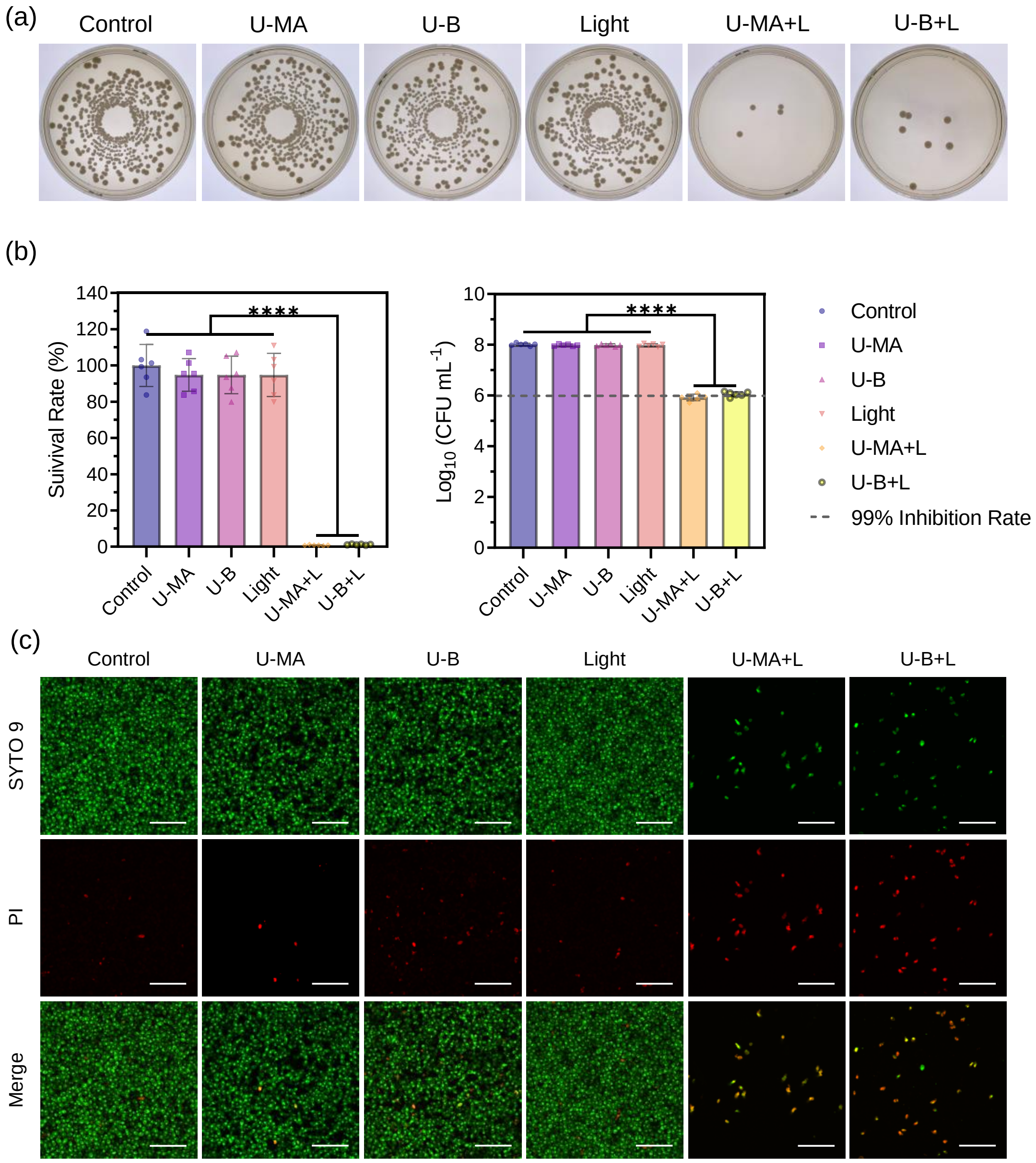

Supplementary Fig 32. Photodynamic inhibition of the formation of *C. albicans* biofilm by U-MA and U-B. *C. albicans* were cultured for 24 hours after treatment. **(a)** Representative images of SDA plates for quantification of *C. albicans* viability. **(b)** Antibacterial efficiency of PDT mediated by U-MA and U-B against *C. albicans*. Error bars = Standard Deviation (n=6 biologically independent samples). One-way ANOVA was performed followed by Tukey's multiple comparisons. ****: $p < 0.0001$. **(c)** *C. albicans* biofilm detected by CLSM. The scale bars are 50 μm. L: Light.

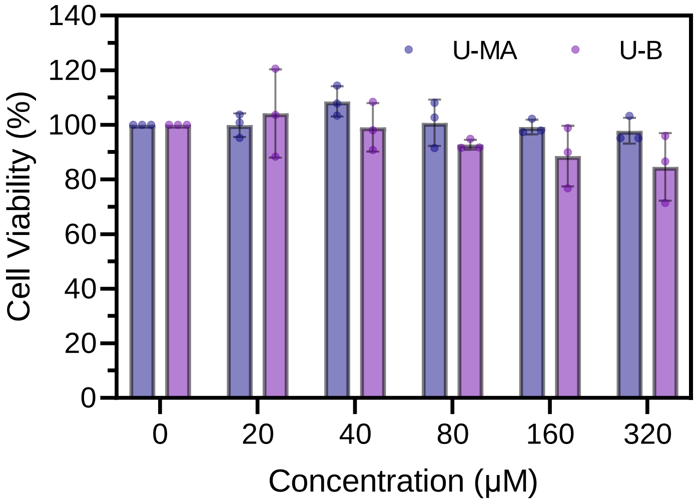

Supplementary Fig 33. Cell viability of HGF cells under treatment with different concentration of U-MA and U-B. Error bars = Standard Deviation (n=3 independent experiments).

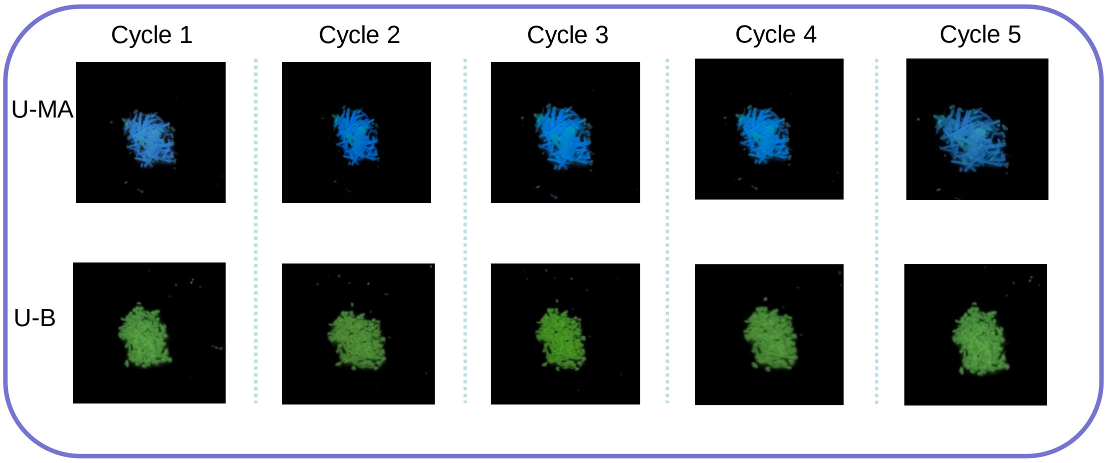

Supplementary Fig 34. Photographs of the phosphorescence of two cocrystals at room temperature and 100℃ cycle.

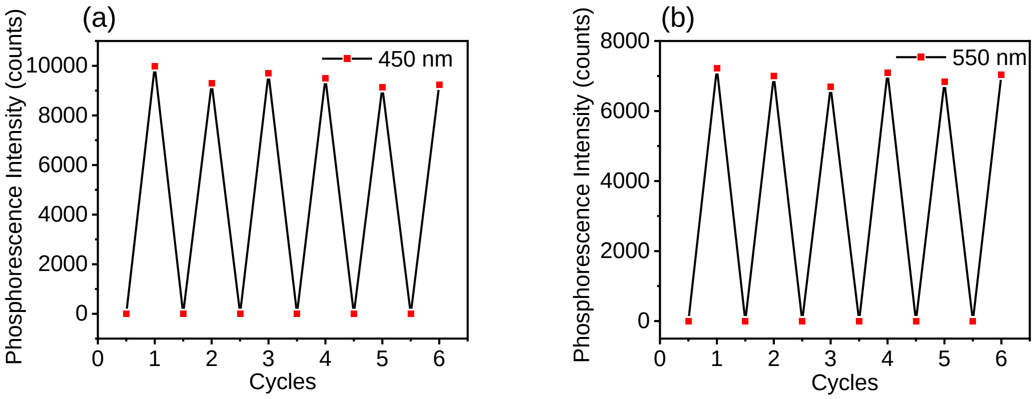

Supplementary Fig 35. The stability of two cocrystals over the temperature cycling of (a) 450 nm of U-MA, (b) 550 nm of U-B.

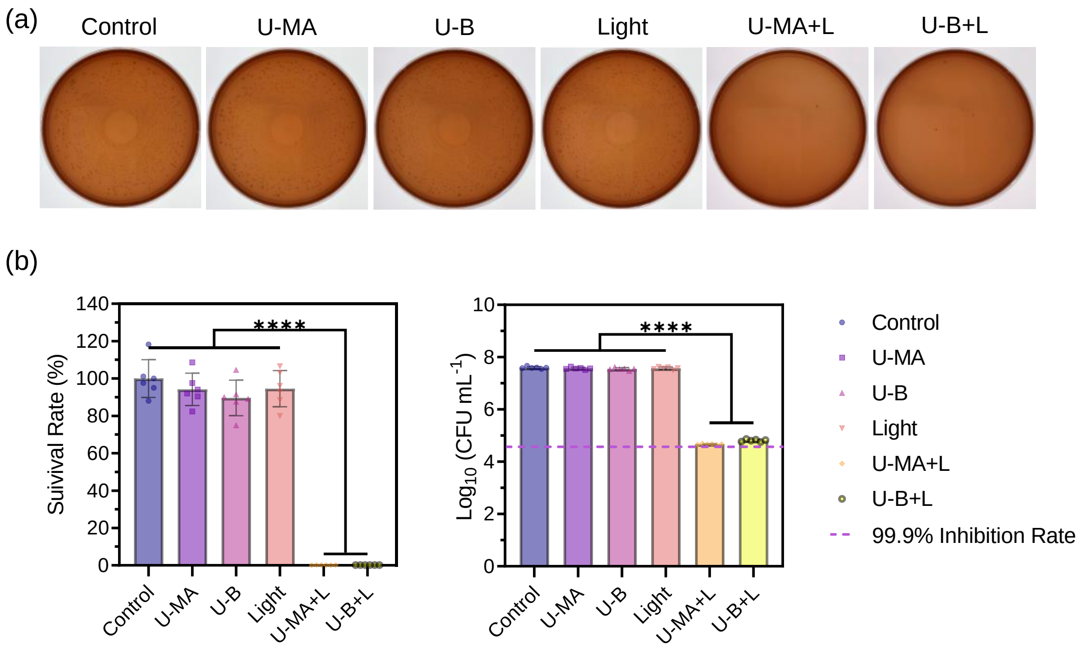

Supplementary Fig 36. Photodynamic inhibition of the formation of *S. mutans* biofilm on dentin surface by U-MA and U-B. Dentin blocks were cultured for 72 hours with *S. mutans* after treatment. **(a)** Representative images of blood agar plates for quantification of *S. mutans* viability. **(b)** Antibacterial efficiency of PDT mediated by U-MA and U-B against *S. mutans*. Error bars = Standard Deviation (n=6 biologically independent samples). One-way ANOVA was performed followed by Tukey's multiple comparisons. ****: $p < 0.0001$. L: Light.

	Microorganism Name	Species	Culture Medium	Agar Plate	Culture Type
Gram-positive (G+)	*S. mutans*	ATCC 35668	BHI broth	Blood agar	anaerobic incubation
	S. aureus	ATCC 29213	M-H broth	Blood agar	aerobic incubation
Gram-negative (G-)	*E. coli*	ATCC 8739	LB	LB agar	aerobic incubation
Fungus	*C. albicans*	ATCC 90028	SDB	SDA	aerobic incubation

BHI broth: Brain Heart Infusion broth; M-H broth: Mueller-Hinton broth; LB: Lysogeny broth; SDB: Sabouraud Dextrose broth; SDA: Sabaurand Dextrose agars.

Supplementary Table 2. The essential information of microorganisms.

Cartesian coordinates of boric acid:

B	0.00873200	-0.03754700	-0.00000800
O	-0.01341600	1.33888700	0.00002000
O	1.21914000	-0.65239400	-0.00014700
O	-1.10056400	-0.83361500	0.00022100
H	-1.94137600	-0.37553300	-0.00124000
H	-0.87768500	1.75199400	0.00001700
H	1.93412500	-0.01174500	0.00051100

Cartesian coordinates of uracil:

O	2.25367500	-1.02086500	0.00029700
N	1.17060100	0.98383600	-0.00080600
H	2.06793800	1.44302200	-0.00075400
O	-2.30145100	-1.00359900	-0.00060100
N	-0.03188900	-0.98483800	0.00018300
H	-0.04193500	-1.99658700	0.00066700
C	0.00040900	1.70128100	0.00010900
H	0.11933000	2.77744400	0.00044200
C	-1.28736800	-0.35218400	0.00029700
C	1.22012800	-0.40388000	-0.00000600
C	-1.20181500	1.10630900	0.00048700
H	-2.12222300	1.66968800	0.00112300

Cartesian coordinates of melamine:

N	-1.15678100	0.75057100	-0.00003800
N	1.22846600	0.62650800	-0.00015800
N	2.21555700	-1.43760000	-0.00018600
H	2.15243200	-2.43920700	0.00029300
H	3.10459000	-0.97195400	0.00075900
N	0.13718500	2.63750800	0.00186600
N	-2.35279600	-1.19994200	-0.00133300
H	-2.39397000	-2.20268000	0.00398400
H	-3.18868900	-0.64454500	0.00373100
N	-0.07162900	-1.37703200	0.00015500
C	0.06704300	1.28877200	0.00017700
C	1.08265200	-0.70241900	-0.00001900
C	-1.14967700	-0.58634500	-0.00013900
H	-0.71061300	3.17455800	-0.00528700

Cartesian coordinates of U-B:

O	-0.97673800	-0.23779600	-0.00236400
N	-1.94977700	-2.29815400	0.00015000
H	-1.01504400	-2.71658300	-0.00072100
O	-5.51165600	-0.47373700	0.00257100
N	-3.24458800	-0.38224900	0.00020300
H	-3.29979600	0.64224700	-0.00033900
C	-3.08320700	-3.06116800	0.00157500
H	-2.91422700	-4.13060200	0.00196500
C	-4.45065300	-1.07416900	0.00180500
C	-1.98366100	-0.92357600	-0.00078900
C	-4.31509700	-2.52007300	0.00242500
H	-5.21175300	-3.12023500	0.00353200
H	-5.93353900	1.28443700	0.00145300
O	-6.08464600	2.24573600	0.00086400
B	-4.96658100	2.99466100	-0.00053300
O	-3.71008600	2.41238100	-0.00119600
H	-3.00653000	3.06697400	-0.00227900
O	-5.02211500	4.36003000	-0.00137300
H	-5.93069100	4.67121100	-0.00083700
O	2.70470000	0.88674300	-0.00144500
N	4.03503900	-0.95970000	0.00029400
H	4.85333600	-0.34594000	0.00103900
O	0.72426800	-3.20691800	-0.00277600
N	1.72787400	-1.16775500	-0.00208000
H	0.80235800	-0.72126600	-0.00278700
C	4.15724400	-2.31948900	0.00073700
H	5.17438600	-2.69094400	0.00190300
C	1.75814600	-2.55627800	-0.00177000
C	2.81316000	-0.32354600	-0.00109000
C	3.08847900	-3.13619400	-0.00020100
H	3.17993800	-4.21124800	0.00013200
H	7.06685100	0.79804200	0.00333700
O	6.12128900	0.97200000	0.00215300
B	5.88690000	2.34333700	0.00118900
O	6.99338900	3.14363100	0.00196300
H	6.75585400	4.07430200	0.00121700
O	4.64548800	2.85365700	-0.00042500
H	3.91981600	2.20574800	-0.00082100
H	1.03612700	3.08368400	-0.00573200
